# Supplementary material for: βIV-spectrin as a stalk cell-intrinsic regulator of VEGF signaling
Source: Nat Commun. 2022 Mar 14;13:1326. doi: 10.1038/s41467-022-28933-1 (PMC8921520; doi:10.1038/s41467-022-28933-1)
Supplement: Supplementary file 1 — Supplementary Information [file 41467_2022_28933_MOESM1_ESM.pdf]

β_{IV}-spectrin as a stalk cell-intrinsic regulator of VEGF signaling

Supplementary Information

Contents:

Supplementary Figure 1. Detection of β_{IV}-spectrin expression in proliferating ECs and loss of function *in vivo*

Supplementary Figure 2. Analysis of E15.5 embryos

Supplementary Figure 3. Quantitative proteomics workflow

Supplementary Figure 4. Characterization of β_{IV}-spectrin-dependent changes in VEGFR2 and EC proliferation in vasculature of developing and mature retina

Supplementary Figure 5. Subcellular distribution of β_{IV}-spectrin and VEGFR2 in ECs

Supplementary Figure 6. β_{IV}-spectrin deficient at CaMKII-binding enhances VEGFR2 cell surface retention

Supplementary Figure 7. Characterization of vascular sprouting and VEGF signaling in retina and primary ECs of $\beta_{IV}qv^{4J}$ mice

Supplementary Figure 8. MS-phosphoproteomic spectral analysis

Supplementary Figure 9. Immunofluorescence staining of P5 WT and β_{IV}-EC^{KO} retina

Supplementary Figure 10. Biochemical analysis of Smad1/5 and Smad2/3 activation

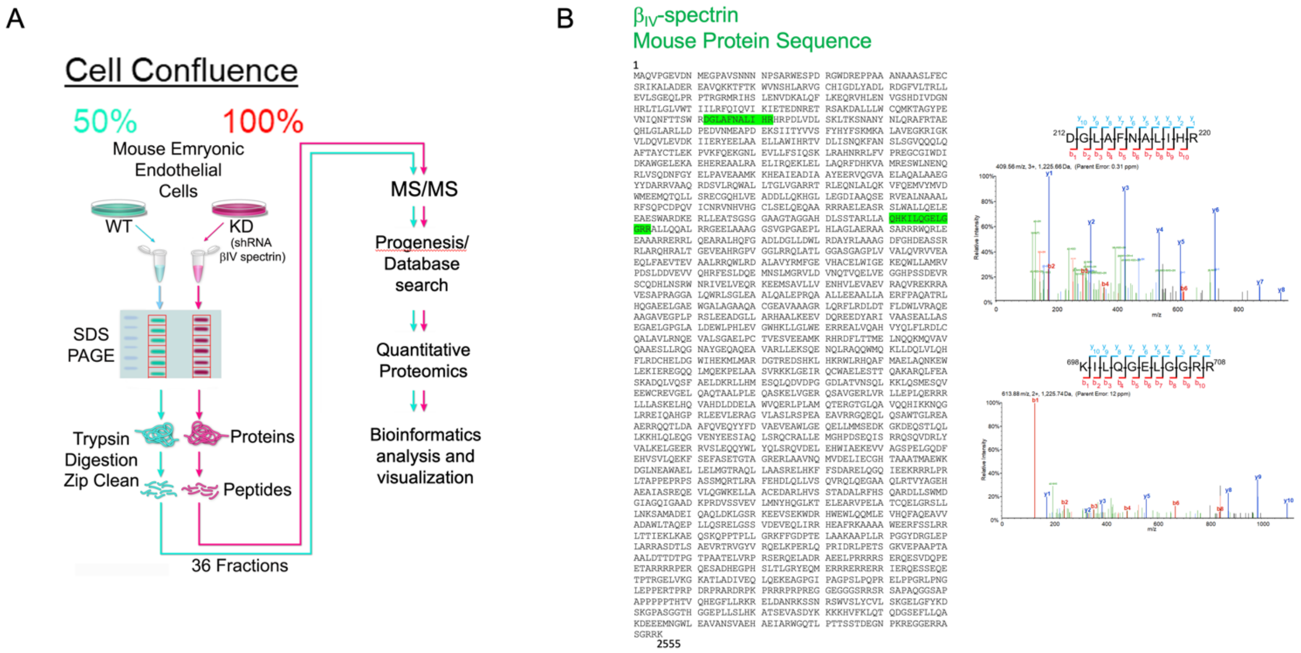

Supplementary Figure 1. Detection of β_{IV}-spectrin expression in proliferating ECs and loss of function *in vivo*.

(A) Schematic shows the work-flow for quantitative proteomics. MEECs were harvested at 50% or 100% confluence (n=3 per group), then normalized for total protein concentration prior to being resolved on SDS-PAGE. Each lane was cut into 5 gel pieces for trypsin digest and MS analysis. (B) MS spectra of two peptide fragments identified corresponded to β_{IV}-spectrin (green highlighted peptide sequence), which were detected in proliferative but not quiescent stage of growth.

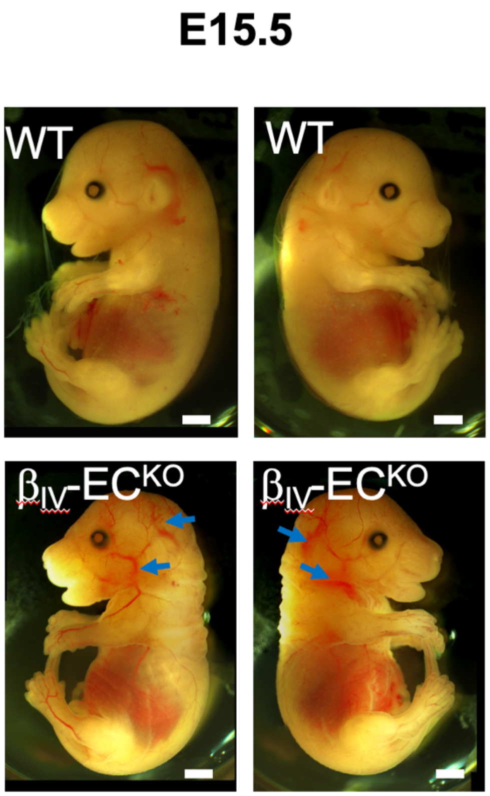

Supplementary Figure 2. Analysis of E15.5 embryos. Images representative of WT and β_{IV}-EC^{KO} embryos harvested at E15.5 upon tamoxifen induction at E8.5. β_{IV}-EC^{KO} feature more dilated vasculature with sporadic bleeding (blue arrows). Scale bar: 1000µm.

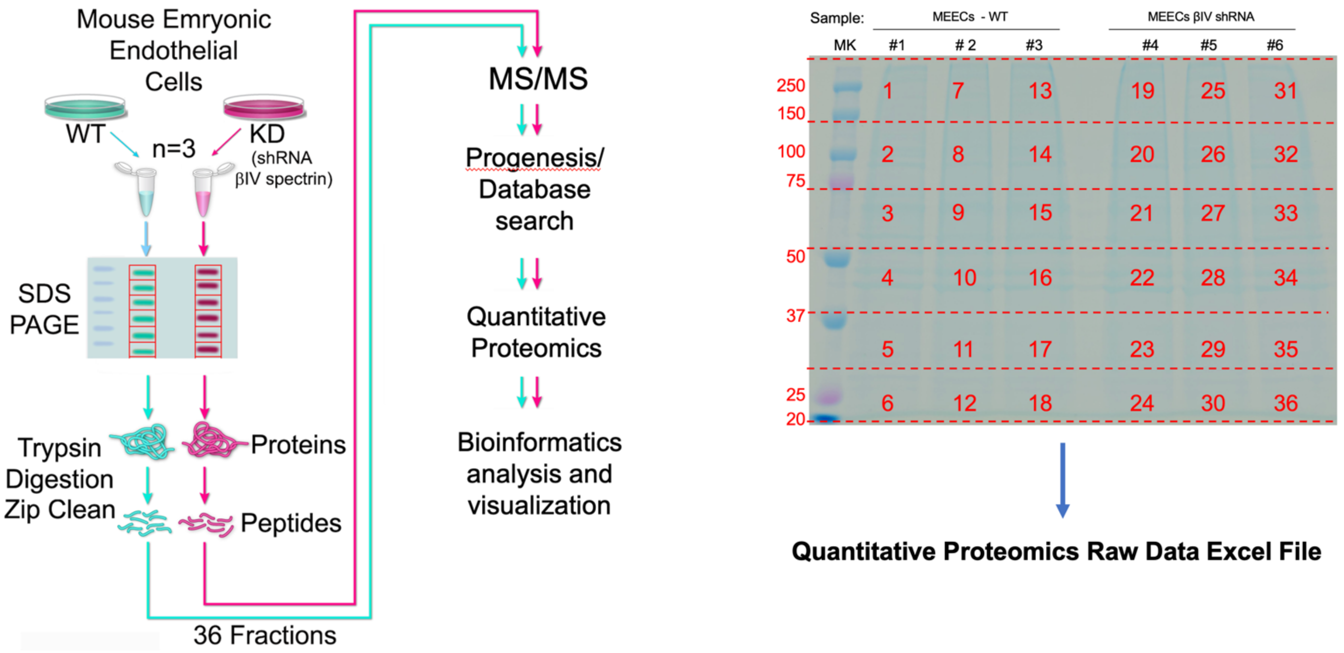

Supplementary Figure 3. Quantitative proteomics workflow. Schematic shows the workflow for quantitative proteomics of WT versus β_{IV}-shRNA MEECs. Quantitative proteomics raw data files are presented separately.

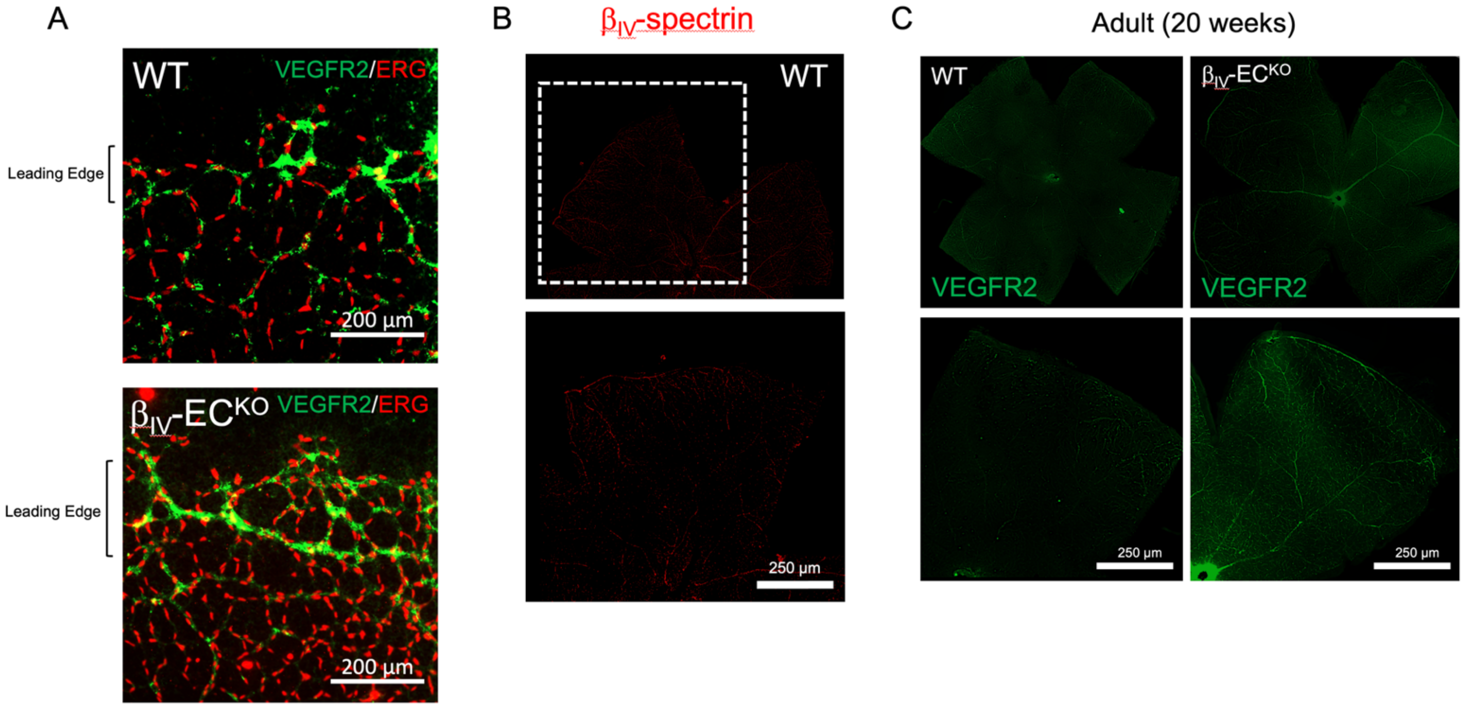

Supplementary Figure 4. Characterization of β_{IV}-spectrin-dependent changes in VEGFR2 and EC proliferation in vasculature of developing and mature retina.

(A) Shown are immunofluorescence images of VEGFR2 (green) and ERG (red) co-staining at P5 of WT and β_{IV}-EC^{KO} retina near the leading edge of vascular expansion.

(B) Images show β_{IV}-spectrin staining in adult WT retina (red).

(C) Shown are representative images of VEGFR2 staining (green) in adult WT and β_{IV}-EC^{KO} retina (20 weeks of age).

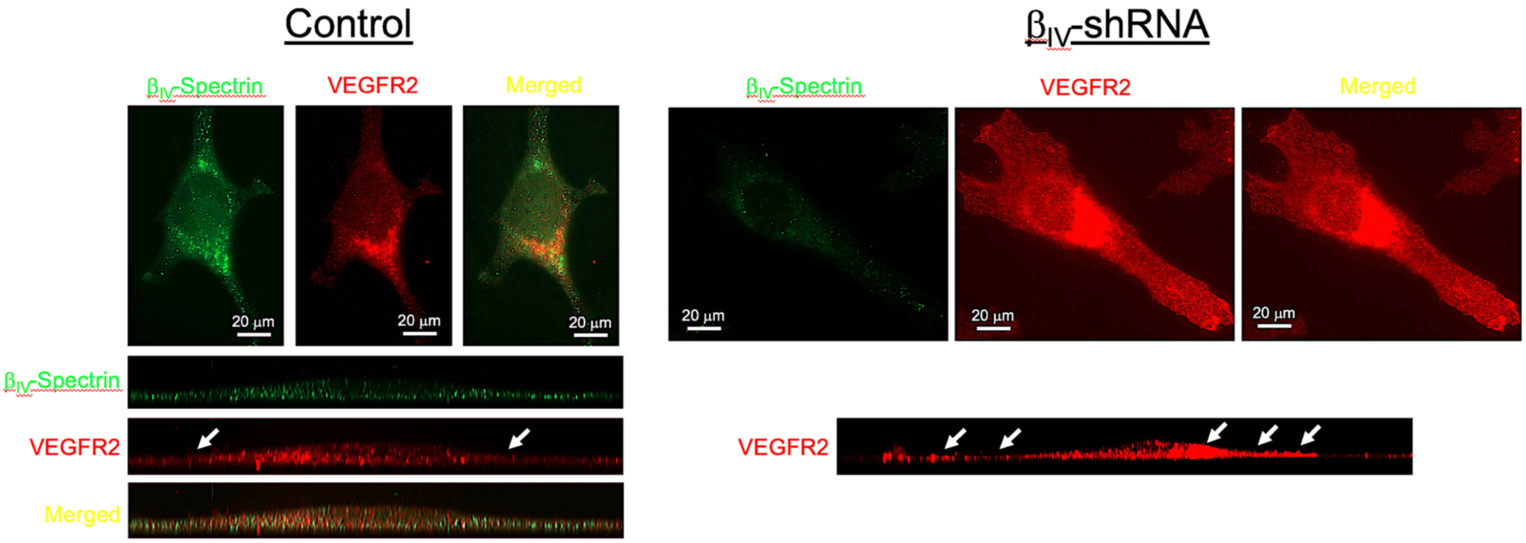

Supplementary Figure 5. Subcellular distribution of β_{IV}-spectrin and VEGFR2 in ECs. Shown are representative 2D and 3D immunofluorescence staining of endogenous β_{IV}-spectrin (green) and VEGFR2 (red) in WT and β_{IV}-shRNA MEECs. Z-stack images and white arrows in lower panels demonstrate the greater overall basolateral accumulation of VEGFR2 in β_{IV}-shRNA cells.

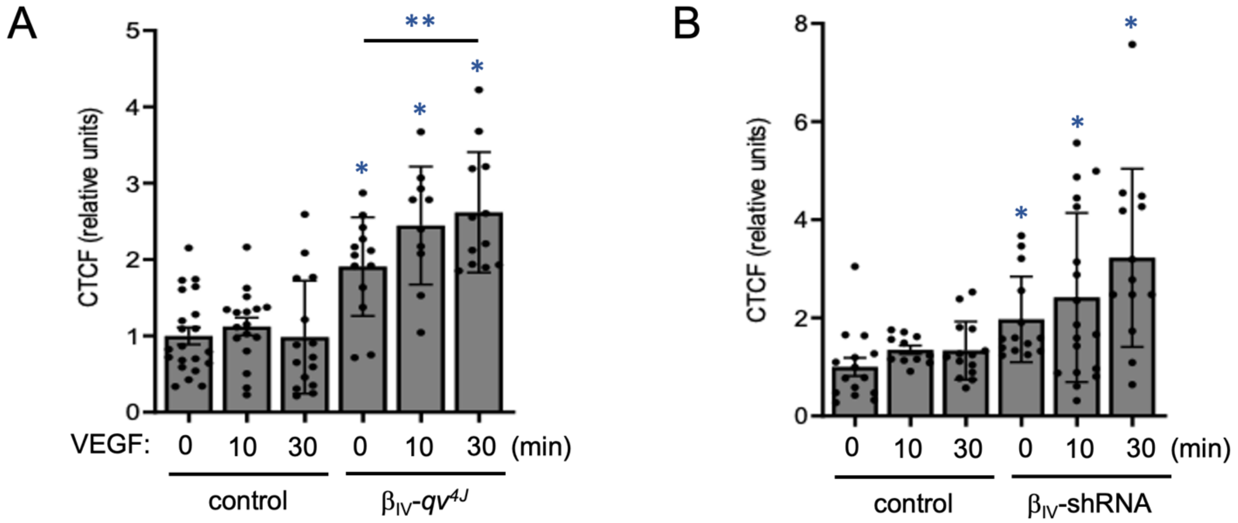

Supplementary Figure 6. β_{IV}-spectrin deficient at CaMKII-binding enhances VEGFR2 cell surface retention.

Graphs represent quantification of cell surface endogenous VEGFR2 levels based on immunofluorescence staining of nonpermeabilized control versus either $\beta_{IV}qv^{4J}$ (A) or β_{IV}-shRNA where a Type 2 t-test results show: *p=0.0007 or lower; **p=0.04 or lower relative to control at 0 min or as indicated. (B) ECs upon stimulation with VEGF at indicated time points. Data representative of three independent experiments with quantification of 25 cells per group per experiment. A Type 2 t-test results show: *p=0.002 or lower relative to control at 0 min. Data are presented as mean values ± SEM.

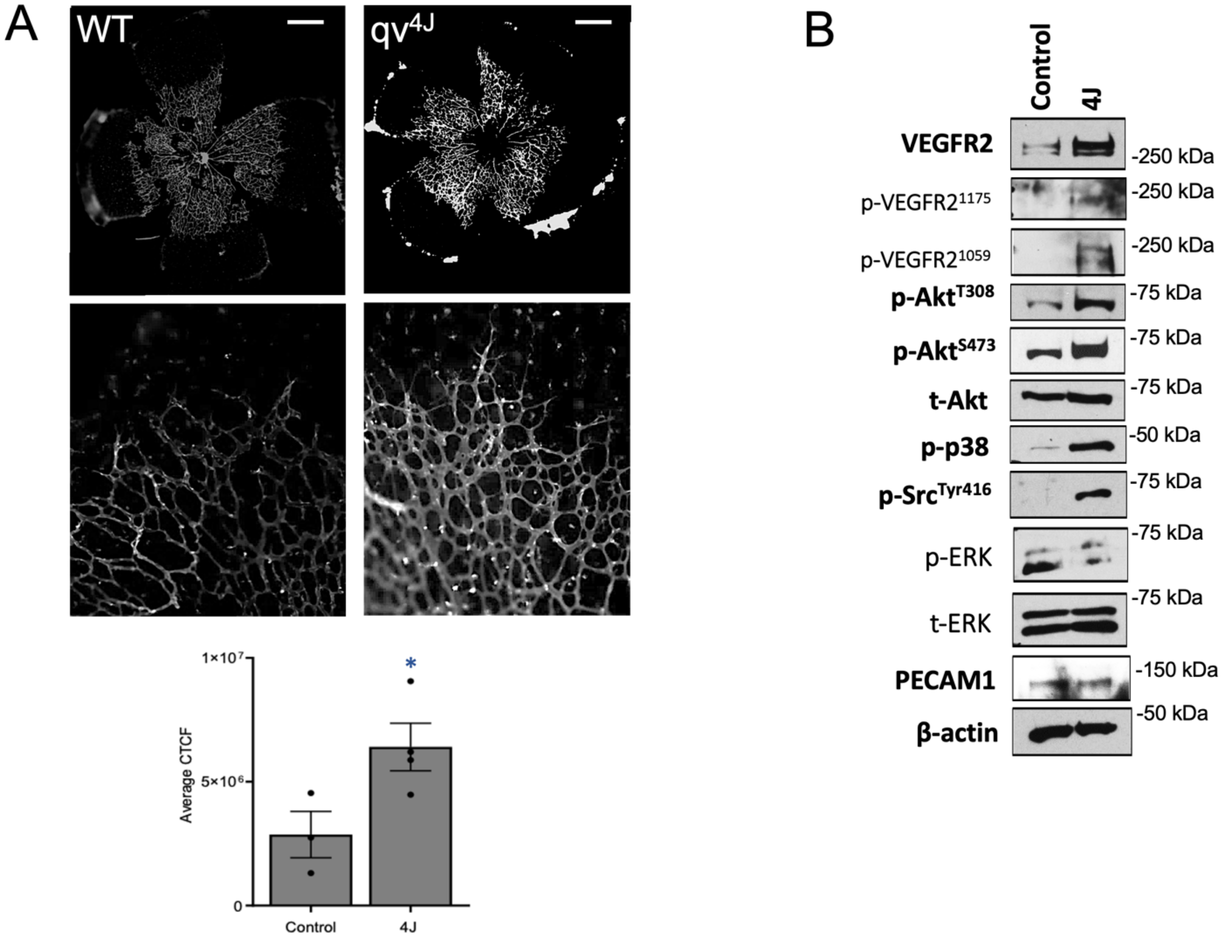

Supplementary Figure 7. Characterization of vascular sprouting and VEGF signaling in retina and primary ECs of $\beta_{IV}qv^{4J}$ mice.

(A) IB4 staining of P5 retina of WT and $\beta_{IV}qv^{4J}$ mice. Graph quantification based mean CTCF values ± SEM. Graphs represent n=3 mice per group. A Type 2 t-test results show: *p=0.02 relative to control. Scale bar: 1000µm.

(B) Biochemical analysis of VEGF signaling in primary ECs isolated from WT and $\beta_{IV}qv^{4J}$ mice.

Mouse VEGFR2 Peptide Sequence

```
1
MESKALLAVA LWFCVETRAA SVGLPGDFLH PPKLSTQKDI LTILANTTLQ
ITCRGQRDLD WLWPNAQRDS EERVLVTECG GGDSIFCKTL TIPRVVGNDT
GAYKCSYRDV DIASTVYVYV RDYRSPFIAS VSDQHGIVYI TENKNKTVVI
PCRGSISNLN VSLCARYPEK RFVPDGNRIS WDSEIGFTLP SYMISYAGMV
FCEAKINDET YQSIMYIVVV VGYRIYDVIL SPPHEIELSA GEKLVLNCTA
RTELNVGLDF TWHSPPSKSH HKKIVNRDVK PFPGTVAKMF LSTLTIESVT
KSDQGEYTCV ASSGRMIKRN RTFVRVHTKP FIAFGSGMKS LVEATVGSQV
RIPVKYLSYP APDIKWYRNG RPIESNYTMI VGDELTIMEV TERDAGNYTV
ILTNPISMEK QSHMVSLVVN VPPQIGEKAL ISPMDSYQYG TMQTLTCTVY
ANPPLHHIQW YWQLEEACSY RPGQTSPYAC KEWRHVEDFQ GGNKIEVTKN
QYALIEGKNK TVSTLVIQAA NVSALYKCEA INKAGRGERV ISFHVIRGPE
ITVQPAAQPT EQESVSLLCT ADRNTFENLT WYKLGSQATS VHMGESLTPV
CKNLDALWKL NGTMFSNSTN DILIVAFQNA SLQDQGDYVC SAQDKKTKKR
HCLVKQLIIL ERMAPMITGN LENQTTTIGE TIEVTCPASG NPTPHITWFK
DNETLVEDSG IVLRDGNRNL TIRRVRKEDG GLYTCQACNV LGCARAETLF
IIEGAQEKTN LEVIILVGTA VIAMFFWLLL VIVLRTVKRA NEGELKTGYL
SIVMDPDELP LDERCERLPY DASKWEFPRD RLKLGKPLGR GAFGQVIEAD
AFGIDKTATC KTVAVKMLKE GATHSEHRAL MSELKILIHI GHHLNVVNLL
GACTKPGGPL MVIVEFCKFG NLSTYLRGKR NEFVPYKSKG ARFRQGKDYV
GELSVDLKRR LDSITSSQSS ASSGFVEEKS LSDVEEEEAS EELYKDFLTL
EHLICYSFQV AKGMEFLASR KCIHRDLAAR NILLSEKNVV KICDFGLARD
IYKDPDYVRK GDARLPLKWM APETIFDRVY TIQSDVWSFG VLLWEIFSLG
ASPYPGVKID EEFCRRLKEG TRMRAPDYTT PEMYQTMLDC WHEDPNQRPS
FSELVEHLGN LLQANAQQDG KDYIVLPMSE TLSMEEDSGL SLPTSPVSCM
EEEEVCDPKF HYDNTAGISH YLQNSKRKSR PVSVKTFEDI PLEEPEVKVI
PDDSQTDSGM VLASEELKTL EDRNKLSPSF GGMMPSKSRE SVASEGSNQT
SGYQSGYHSD DTDTTVYSSD EAGLLKMVDA AVHADSGTTL RSPPV
                                                 1345
```

Mouse VEGFR2 Phosphopeptide Analysis

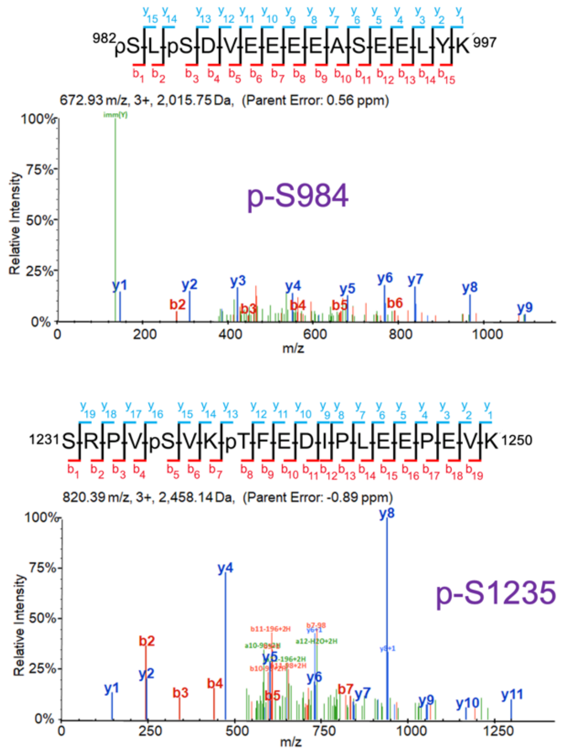

Supplementary Figure 8. MS-phosphoproteomic spectral analysis. Shown are mouse VEGFR2 protein sequence with phosphopeptide sequences highlighted (green). Representative spectral peaks indicate phosphorylation signals present on S984 and S1235 in VEGFR2 of WT but not in β_{IV}-shRNA MEECs.

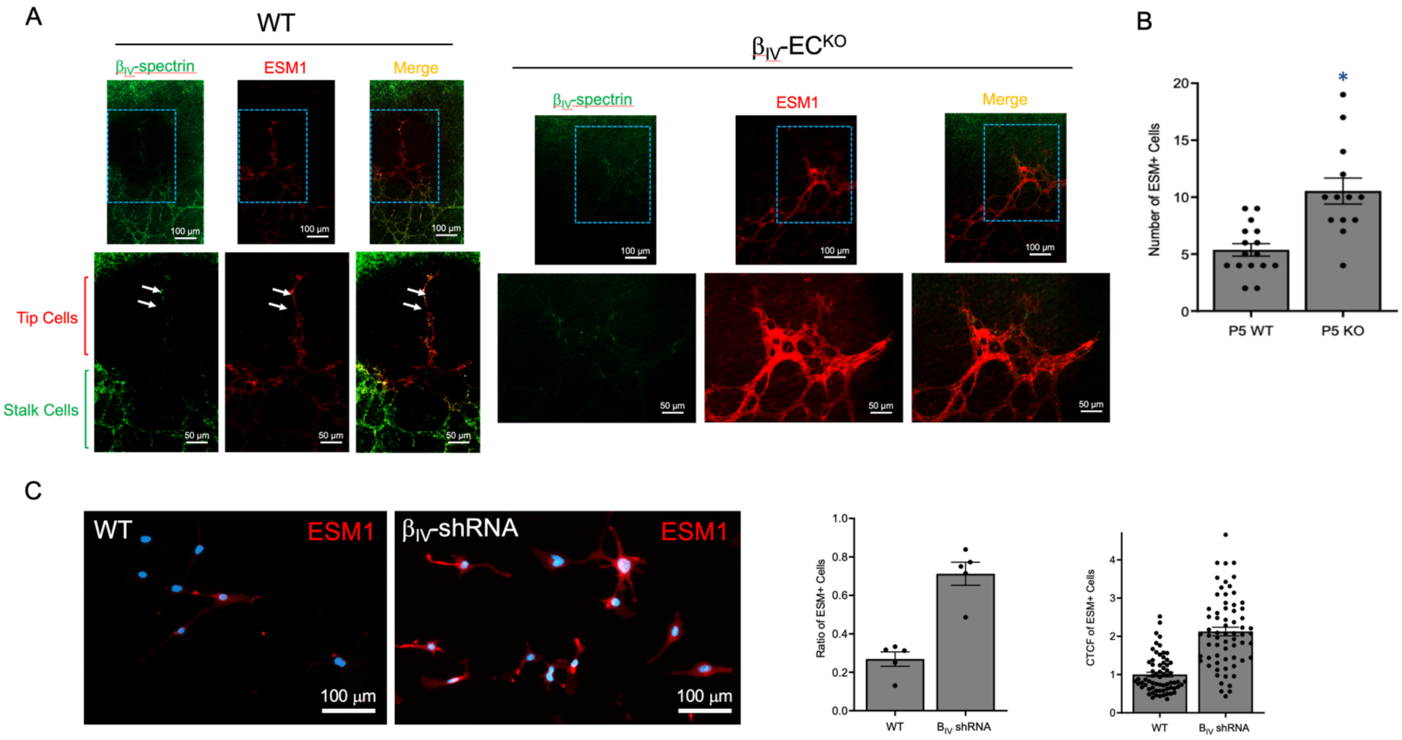

Supplementary Figure 9. Immunofluorescence staining of P5 WT and β_{IV}-EC^{KO} retina.

(A) Shown are representative confocal fluorescence images of β_{IV}-spectrin (green) and EC tip marker ESM1 (red). Magnified images have white arrows indicating ESM1 staining at the leading edge of the vascular expansion. Merged image in WT shows that β_{IV}-spectrin expression is excluded from the ESM1 expressing tip cell and vice versa. In β_{IV}-EC^{KO} retina there is greater ESM1 expression throughout the leading edge of the vascular expansion upon β_{IV}-spectrin depletion.

(B) Graph quantification is based on ESM1-positive cell counts at the leading edge per lobe of n=16 and 13 for P5 WT and P5 KO respectively for 3 separate mouse retinas. A Type 2 t-test results show: *p=0.0003 relative to P5 WT.

(C) Fluorescence images show co-staining of ESM (red) and DAPI (blue) in WT control and B_{IV}-shRNA MEECs. Graph quantifications represent ratio of ESM-positive ECs as a mean value ± SEM, where n= 67 and 66 cells for WT and B_{IV}-shRNA respectively. A Type 2 t-test results show: *p=3.96E^{-15} relative to WT for CTCF and *p=0.0002 compared to WT for ESM+ cells.

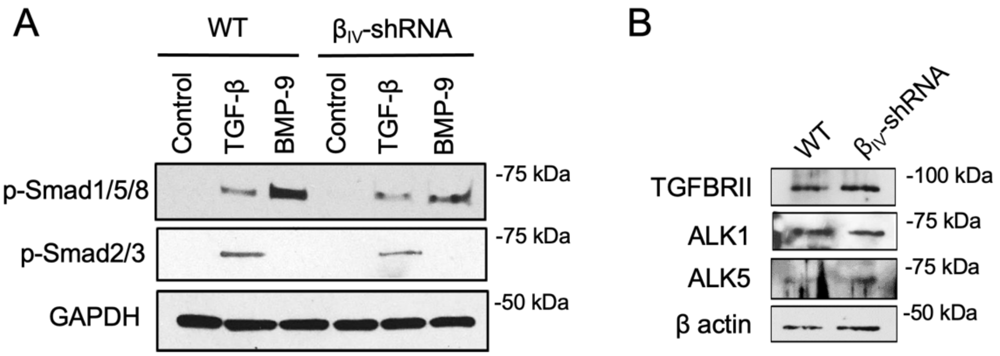

Supplementary Figure 10. Biochemical analysis of Smad1/5 and Smad2/3 activation.
(A) Western analysis shows phospho-Smad1/5/8 and phospho-Smad2/3 levels in WT and β_{IV}-shRNA ECs upon serum deprivation for 5 h prior to stimulation with TGF-β (200 pM) or BMP9 (1 ng/mL) for 30 min.
(B) Western shows the levels of the TGF-β receptors that mediate Smad1/5 and Smad2/3 signaling in ECs including ALK1, ALK5 and TGFβRII.
