# Supplementary material for: βIV-spectrin as a stalk cell-intrinsic regulator of VEGF signaling
Source: Nat Commun. 2022 Mar 14;13:1326. doi: 10.1038/s41467-022-28933-1 (PMC8921520; doi:10.1038/s41467-022-28933-1)
Supplement: Supplementary file 3 — Description of Additional Supplementary Files [file 41467_2022_28933_MOESM3_ESM.pdf]

Description of Additional Supplementary Files

Title: Supplementary Data 1

Description: A key resources table lists the antibodies, chemicals and reagents used in the study.
