# Supplementary material for: βIV-spectrin as a stalk cell-intrinsic regulator of VEGF signaling
Source: Nat Commun. 2022 Mar 14;13:1326. doi: 10.1038/s41467-022-28933-1 (PMC8921520; doi:10.1038/s41467-022-28933-1)
Supplement: Supplementary file 4 — Supplementary Data 1 [file 41467_2022_28933_MOESM4_ESM.pdf]

KEY RESOURCES TABLE

REAGENT or RESOURCE	SOURCE	IDENTIFIER
Antibodies		
Spectrin β_{IV} Antibody<br>1:1000 dilution	Santa Cruz Biotechnology<br>StressMarq Biosciences	Product #. sc-514744<br>S393-29
VEGF Receptor 2 Antibody<br>1:1000 dilution	Cell Signaling Technology<br>Santa Cruz Biotechnology	Product #. 9698S<br>sc-6251
Phospho-VEGF Receptor 2 (Tyr996)<br>1:1000 dilution	Cell Signaling Technology	Product #.2474S
Phospho-VEGF Receptor 2 (Tyr1059)<br>1:1000 dilution	Cell Signaling Technology	Product # 3817S
Phospho-VEGF Receptor 2 (Tyr1175)<br>1:1000 dilution	Cell Signaling Technology	Product #.3770S
Phospho-Akt (Thr308)<br>1:1000 dilution	Cell Signaling Technology	Product #.13038S
Phospho-Akt (Ser473)<br>1:1000 dilution	Cell Signaling Technology	Product #.4060S
Phospho-Src Family (Tyr416)<br>1:1000 dilution	Cell Signaling Technology	Product #.6943S
Phospho-p44/42 MAPK (Erk1/2) (Thr202/Tyr204)<br>1:1000 dilution	Cell Signaling Technology	Product #.9101S
p44/42 MAPK (Erk1/2)<br>1:1000 dilution	Cell Signaling Technology	4695S
Phospho-p38 MAPK (Thr180/Tyr182)<br>1:1000 dilution	Cell Signaling Technology	Product #.3871S
Phospho-PLCγ2 (Tyr1217) Antibody<br>1:1000 dilution	Cell Signaling Technology	Product #.4511S
Phospho-eNOS (Ser1177)<br>1:1000 dilution	Cell Signaling Technology	Product #.9570S
Anti-β-Actin antibody<br>1:4000 dilution	Sigma-Aldrich	Product #.A1978
CaMKII Antibody<br>1:500 dilution	Santa Cruz Biotechnology	Product #.sc-9035
Anti-CD31 antibody<br>1:1000 dilution	Abcam	Product #.ab7388
Anti-CD34 antibody<br>1:1000 dilution	Abcam	Product #.ab81289
Dll4<br>1:1000 dilution	Cell Signaling Technology	Product #.2558

Notch 1 Antibody<br>1:500 dilution	Santa Cruz Biotechnology	Product #.sc-9170
Jagged1 Antibody<br>1:500 dilution	Santa Cruz Biotechnology	Product #.sc-8303
Anti-ERG antibody<br>1:1000 dilution	Abcam	Product #.ab92513
Bacterial and virus strains		
Biological samples		
Chemicals, peptides, and recombinant proteins		
	Sigma-Aldrich	Product #.
KN-93	Sigma-Aldrich	Product #.K1385
Lipofectamine 2000 Transfection Reagent	Thermo Fisher Scientific	Product #.11668027
Polybrene	Santa Cruz Biotechnology	Product #.Sc-134220
EZ-Link™ Sulfo-NHS-LC-Biotin	Thermo Fisher Scientific	Product #.21335
Streptavidin beads	Cell Signaling Technology	Product #.9419S
Pierce Peptide Desalting Spin Columns	Thermo Fisher Scientific	Product # 89852
High-Select Fe-NTA Phosphopeptide Enrichment Kit	Thermo Fisher Scientific	Product # A32992
High-Select TiO2 Phosphopeptide Enrichment Kit	Thermo Fisher Scientific	Product # A32993
Critical commercial assays		
Deposited data		
ProteomeXchange accession code PXD026618		

Experimental models: cell lines		
Human microvascular endothelial cell-1 (HMEC-1)	ATCC	#CRL-3243
Human umbilical vein endothelial cells (HUVEC)	ATCC	Pooled PCS-100-013
Murine embryonic endothelial cells (MEEC)	Immortalized cells from mouse embryos- custom	
Mouse Aortic Endothelial Cells (MAEC)	Primary cells from normal mice	
Experimental models: organisms/strains		
Fli1-EGFP		
Mouse qv4J	Jackson Laboratories	
Mouse β_{IV}-Spectrin Fl/Fl	Custom Made	
Oligonucleotides		
Recombinant DNA		
Control shRNA Plasmid	Santa Cruz Biotechnology	Product #.sc-108080
Spectrin β IV shRNA Plasmid (mouse) lentivirus	Santa Cruz Biotechnology	Product #.sc-153737-SH
Plasmid: CamKII-GFP	Addgene	
Plasmid: VEGFR2 WT	Gift from Gerard Blobe	
Plasmid: VEGFR2 S984A		
Plasmid: VEGFR2 S1235A		
Spectrin β IV shRNA Plasmid (mouse)	Sigma Aldrich Mission shRNA	TRCN0000426895 TRCN0000091515
Spectrin β IV shRNA Plasmid (human)	Sigma Aldrich Mission shRNA	TRCN0000113940 TRCN0000413540
Software and algorithms		
ImageJ		
Adobe Photoshop		
Other		
