# Supplementary material for: βIV-spectrin as a stalk cell-intrinsic regulator of VEGF signaling
Source: Nat Commun. 2022 Mar 14;13:1326. doi: 10.1038/s41467-022-28933-1 (PMC8921520; doi:10.1038/s41467-022-28933-1)
Supplement: Supplementary file 5 — Reporting Summary [file 41467_2022_28933_MOESM5_ESM.pdf]

nature portfolio

Corresponding author(s): Nam Y. Lee

Last updated by author(s): 2022/01/31

Reporting Summary

Nature Portfolio wishes to improve the reproducibility of the work that we publish. This form provides structure for consistency and transparency in reporting. For further information on Nature Portfolio policies, see our Editorial Policies and the Editorial Policy Checklist.

Statistics

For all statistical analyses, confirm that the following items are present in the figure legend, table legend, main text, or Methods section.

n/a	Confirmed	
☐	☒	The exact sample size (*n*) for each experimental group/condition, given as a discrete number and unit of measurement
☐	☒	A statement on whether measurements were taken from distinct samples or whether the same sample was measured repeatedly
☐	☒	The statistical test(s) used AND whether they are one- or two-sided<br>*Only common tests should be described solely by name; describe more complex techniques in the Methods section.*
☒	☐	A description of all covariates tested
☒	☐	A description of any assumptions or corrections, such as tests of normality and adjustment for multiple comparisons
☐	☒	A full description of the statistical parameters including central tendency (e.g. means) or other basic estimates (e.g. regression coefficient) AND variation (e.g. standard deviation) or associated estimates of uncertainty (e.g. confidence intervals)
☐	☒	For null hypothesis testing, the test statistic (e.g. *F*, *t*, *r*) with confidence intervals, effect sizes, degrees of freedom and *P* value noted<br>*Give P values as exact values whenever suitable.*
☒	☐	For Bayesian analysis, information on the choice of priors and Markov chain Monte Carlo settings
☒	☐	For hierarchical and complex designs, identification of the appropriate level for tests and full reporting of outcomes
☐	☒	Estimates of effect sizes (e.g. Cohen's *d*, Pearson's *r*), indicating how they were calculated

Our web collection on statistics for biologists contains articles on many of the points above.

Software and code

Policy information about availability of computer code

Data collection	Proteomics data were collected using Xcalibur 2.3 (Thermo Sci). Tandem mass spectra were extracted from Xcalibur RAW files and charge states were assigned using ProteoWizard 3.0.1 msConvert script
Data analysis	Scaffold 5.1. was used to validate MS/MS peptide and protein identifications. Protein probabilities were assigned via Protein Prophet algorithm. Progenesis 2.4 was used for ion intensity quantification. ImageJ was used for image analysis. Excel and Prism were used for graphing and statistical analyses.

For manuscripts utilizing custom algorithms or software that are central to the research but not yet described in published literature, software must be made available to editors and reviewers. We strongly encourage code deposition in a community repository (e.g. GitHub). See the Nature Portfolio guidelines for submitting code & software for further information.

Data

Policy information about availability of data

All manuscripts must include a data availability statement. This statement should provide the following information, where applicable:

- Accession codes, unique identifiers, or web links for publicly available datasets
- A description of any restrictions on data availability
- For clinical datasets or third party data, please ensure that the statement adheres to our policy

The proteomics data generated in this study have been deposited in the ProteomeXchange via the PRIDE database under accession code PXD026618 []. The proteomics data generated in this study are provided in the Supplementary Information/Source Data file.

Field-specific reporting

Please select the one below that is the best fit for your research. If you are not sure, read the appropriate sections before making your selection.

[x] Life sciences   [ ] Behavioural & social sciences   [ ] Ecological, evolutionary & environmental sciences

For a reference copy of the document with all sections, see nature.com/documents/nr-reporting-summary-flat.pdf

Life sciences study design

All studies must disclose on these points even when the disclosure is negative.

Sample size	Sample sizes are indicated in the figure legends. Each experiment was performed a minimum of three times, each as a biologically independent experiment.
Data exclusions	No data was excluded
Replication	Each experiment involved n=3 independent experiments, each involving triplicate or more of samples per group
Randomization	Randomization was performed on capturing regions of interest (ROIs) in fluorescence image analyses. In other experiments, rigorous and unbiased quantifications were performed using densitometry scans (westerns) and imageJ software.
Blinding	Blinding was not performed as the present work did not involve animal behavioral work or scoring by a pathologist. Instead, all molecular and biochemical data analyses were performed using unbiased densitometry scanning, fluorescence readouts, and/or randomized analysis of ROIs during imaging.

Reporting for specific materials, systems and methods

We require information from authors about some types of materials, experimental systems and methods used in many studies. Here, indicate whether each material, system or method listed is relevant to your study. If you are not sure if a list item applies to your research, read the appropriate section before selecting a response.

Materials & experimental systems

n/a	Involved in the study	
[ ]	[x]	Antibodies
[ ]	[x]	Eukaryotic cell lines
[x]	[ ]	Palaeontology and archaeology
[ ]	[x]	Animals and other organisms
[x]	[ ]	Human research participants
[x]	[ ]	Clinical data
[x]	[ ]	Dual use research of concern

Methods

n/a	Involved in the study	
[x]	[ ]	ChIP-seq
[x]	[ ]	Flow cytometry
[x]	[ ]	MRI-based neuroimaging

Antibodies

Antibodies used	All items have been listed in the KEY RESOURCES TABLE
Validation	All items have been validated by our group and others including commercial vendors or researchers

Eukaryotic cell lines

Policy information about cell lines

Cell line source(s)	MEECs were previously derived from mouse embryonic endothelial cells and immortalized. This line has been extensively published by our group and others. HMEC1 was purchased from ATCC. All other cells were primary isolated endothelial cells.
Authentication	All cell lines were purchased by ATCC or rigorously validated and published previously.
Mycoplasma contamination	All cells had been Plasmocin-treated and verified negative for mycoplasma.
Commonly misidentified lines (See ICLAC register)	None of the commonly misidentified lines were used.

Animals and other organisms

Policy information about studies involving animals; ARRIVE guidelines recommended for reporting animal research

Laboratory animals	Mice were C57/Bl6 background, with both male and female mice being used at either embryonic (E12.5, E15.5) or postnatal day 5 (P5).
Wild animals	Wild animals were not used in the study
Field-collected samples	Field-collected samples were not used in the study.
Ethics oversight	All animal procedures were performed in accordance with the guidelines approved by the University of Arizona Institutional Animal Care and Use Committee.

Note that full information on the approval of the study protocol must also be provided in the manuscript.
